# Paneth Cells in Intestinal Homeostasis and Tissue Injury

**DOI:** 10.1371/journal.pone.0038965

**Published:** 2012-06-20

**Authors:** Sabrina Roth, Patrick Franken, Andrea Sacchetti, Andreas Kremer, Kurt Anderson, Owen Sansom, Riccardo Fodde

**Affiliations:** 1 Department of Pathology, Josephine Nefkens Institute, Erasmus MC, Rotterdam, The Netherlands; 2 Bioinformatics, Erasmus MC, Rotterdam, The Netherlands; 3 Beatson Institute, Glasgow, Scotland, United Kingdom; National Cancer Institute, United States of America

## Abstract

Adult stem cell niches are often co-inhabited by cycling and quiescent stem cells. In the intestine, lineage tracing has identified *Lgr5* ^+^ cells as frequently cycling stem cells, whereas *Bmi1* ^+^, *mTert* ^+^, *Hopx* ^+^ and *Lrig1* ^+^ cells appear to be more quiescent. Here, we have applied a non-mutagenic and cell cycle independent approach to isolate and characterize small intestinal label-retaining cells (LRCs) persisting in the lower third of the crypt of Lieberkühn for up to 100 days. LRCs do not express markers of proliferation and of enterocyte, goblet or enteroendocrine differentiation, but are positive for Paneth cell markers. While during homeostasis, LR/Paneth cells appear to play a supportive role for *Lgr5* ^+^ stem cells as previously shown, upon tissue injury they switch to a proliferating state and in the process activate *Bmi1* expression while silencing Paneth-specific genes. Hence, they are likely to contribute to the regenerative process following tissue insults such as chronic inflammation.

## Introduction

The dichotomy of cycling and quiescent stem cells is thought to meet the demands of two distinct settings, namely daily turnover and regeneration upon tissue insults [1], [2]. Accordingly, stem cell niches such as skin [3], [4], [5], [6], the stomach antrum [7], [8], and bone marrow [9], [10] have been shown to encompass both quiescent and cycling populations. Whereas cycling stem cells maintain daily homeostasis, their quiescent equivalents have been postulated to play a rate-limiting role in tissue regeneration upon injury [10].

The epithelial lining of the upper gastro-intestinal tract is characterized by a unique tissue architecture consisting of villi and crypts. The intestinal crypt of Lieberkühn is a highly dynamic niche with stem cells residing in its lower third, a position from where they give rise to a population of fast-cycling transit-amplifying (TA) cells. TA cells undergo a limited number of cell divisions and eventually differentiate into the four specialized cell types of the small intestine, namely absorptive, enteroendocrine, goblet, and Paneth cells. Based on clonal analysis and knock-in experiments, it was shown that the crypt base columnar cells (CBCs) located in the lower third of the crypt and earmarked by *Lgr5* (*leucine-rich repeat containing G protein-coupled receptor 5*) expression, represent actively cycling stem cells capable of giving rise to all differentiated cell types of the intestinal epithelium [11], [12], [13], [14]. However, targeted ablation of *Lgr5^+^* CBCs demonstrated that these cells are in fact dispensable [15], [16]. Accordingly, upon radiation-induced tissue injury, *Lgr5* ^+^ CBCs are lost without affecting the regenerative process [17], thus pointing at the existence of additional, possibly quiescent, stem cell types in the intestine capable of underlying tissue regeneration following major tissue injury.

To date, different populations of quiescent or infrequently dividing stem cells have been identified in the mouse intestine by lineage tracing: *Bmi1* was identified as a marker of slower cycling intestinal stem cells located at position +4 from the base of the crypt in the proximal mouse duodenum [18]. Subsequently, cells earmarked by mouse *telomerase reverse transcriptase* expression (*mTert* ^+^) were also found to represent slow cycling stem cells located at position four from the crypt base [19]. The location of *Bmi1* ^+^ and *mTert* ^+^ cells along the crypt-villus axis is reminiscent of cells with long-term BrdU label-retaining capacity, an alleged characteristic of quiescent stem cells, previously shown to map at the same +4 position [20]. Upon irradiation, the +4 cells enter the cell cycle and, as such, are thought to underlie the clonogenic regenerative response of the crypt [21], [22]. Yet, these early studies were limited by the lack of specific markers to allow isolation and lineage tracing of the +4 cells. Moreover, DNA-labeling by BrdU is dependent on cell division and does in itself represent a genotoxic insult for quiescent stem cells, thus triggering their cell cycle activation [9].

The *Dcamkl1* (*doublecortin and CaM kinase-like-1*) was also proposed as a putative intestinal marker of radiation-resistant stem cells though, formally, the lineage labeling capacity of *Dcamkl1* ^+^ cells has not been demonstrated yet [23].

More recent studies added on the complexity of stem cells subpopulations co-inhabiting the intestinal crypt and their inter-relation. First, while the actively cycling *Lgr5* ^+^ CBCs are responsible for daily homeostasis, upon tissue insults their more quiescent *Bmi1* ^+^ and *mTert* ^+^ equivalents at position +4 become active and can give rise to *Lgr5* ^+^ cells and to all differentiated intestinal cell types [19], [24]. Hence, although functionally distinct, cycling and quiescent intestinal stem cells appear to be able to give rise to each other, thus demonstrating a bidirectional lineage relationship [19], [24]. The latter was shown to be true for yet another subpopulation of slow-cycling intestinal stem cells located at position +4 earmarked by expression of *Hopx*, an atypical homeobox protein [25].

Lastly, the pan-ErbB inhibitor *Lrig1* (*Leucine-rich repeats and immunoglobulin-like domain 1*) was elegantly shown to mark non-cycling, long-lived stem cells located at the crypt base which, upon tissue insults, proliferate and divide to underlie the regenerative process [26]. However, the lineage and epistatic relationship among the quiescent SC populations identified by *Bmi1*, *Tert*, *Hopx*, and *Lrig1* is at present unclear.

Here, we have applied a non-mutagenic and cell cycle independent approach to identify label-retaining cells (LRCs) persisting in the lower third of the crypt of Lieberkühn of the mouse small intestine for up to 100 days. These LRCs appear to largely overlap with Paneth cells. Notably, upon radiation-induced tissue injury, LRCs are activated to exit dormancy, enter the cell cycle while progressively losing their Paneth cell identity and acquiring gene expression features reminiscent of stem cells.

## Results

### *In vivo* Identification and Isolation of Infrequently Cycling Intestinal Cells

**Figure 1:**
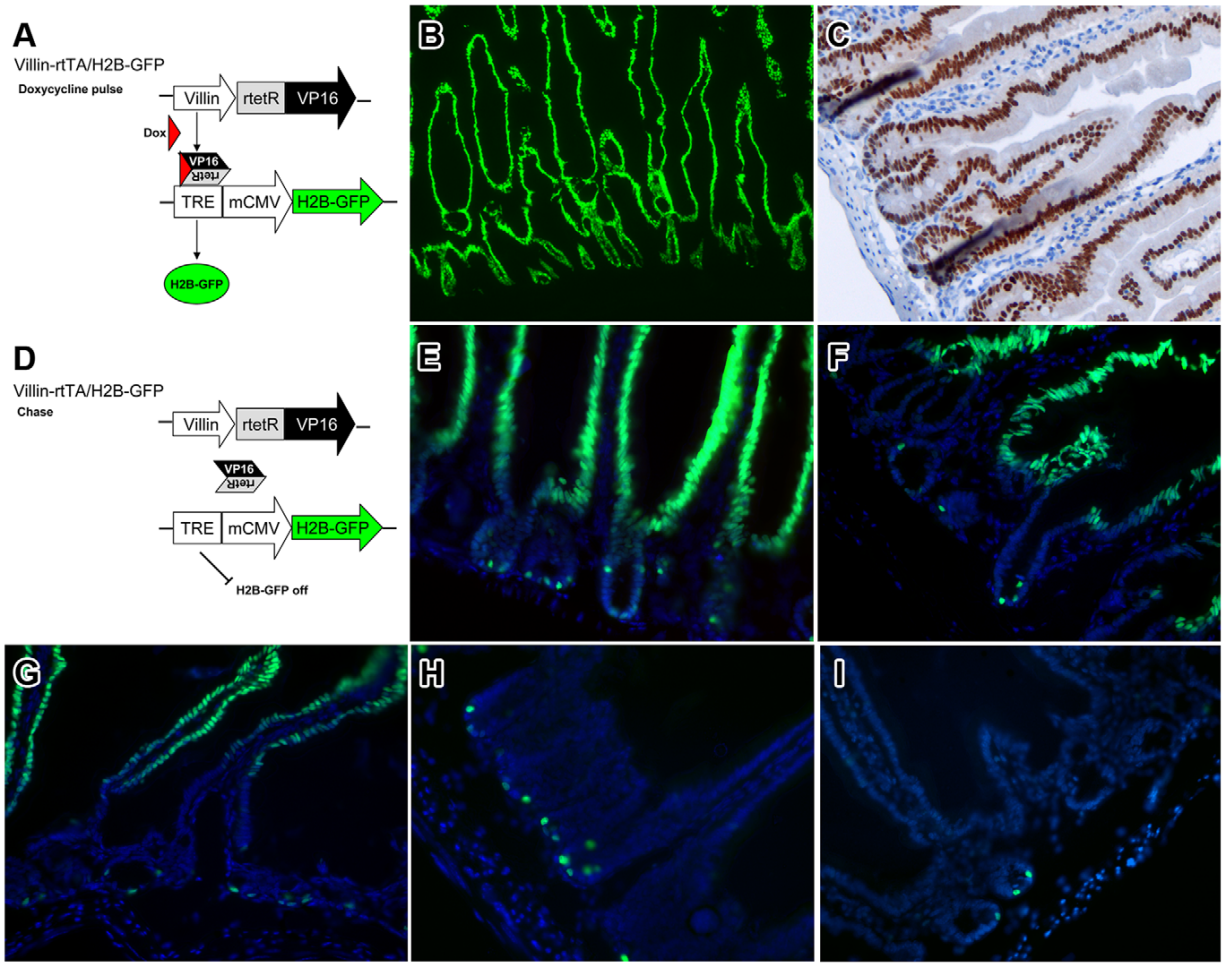
*In vivo* identification and isolation of infrequently cycling cells (LRCs) from the mouse intestine. (A,D) General strategy. Villin-rtTA and TRE-H2B-GFP transgenic mice were bred to generate compound experimental animals. The H2B-GFP construct is exclusively expressed upon doxycycline administration in the drinking water. (B–I) Duodenal sections of villin-rtTA/TRE-H2B-GFP mice before (B,C) and after 2 (E), 4 (F), 7 (G), 10 (H), and 70 (I) days of chase shown by epifluorescence (B, E–I; green), DAPI counterstain (E–I; blue) and by immunohistochemistry (IHC, C, brown). (B,C) Cell labeling occurs throughout the entire crypt-villus axis. (D–I) Following doxycycline-withdrawal, H2B-GFP expression is silenced and progressively lost from dividing cells.

We employed a non-mutagenic and cell cycle independent approach to isolate quiescent label-retaining intestinal cells, namely *in vivo* pulse-chase with the histone 2B - green fluorescent protein (H2B-GFP) [3], [9], [10]. This system compares favorably with BrdU as labeling of cells occurs independently of the cell cycle and viable cells can be recovered for analysis [9]. To this aim, we have adapted the method originally developed by the Fuchs laboratory [3] to label and isolate quiescent skin cells by breeding our transgenic model expressing the tet repressor–VP16 cassette under control of the villin promoter (villin-rtTA) [27] with transgenic animals carrying the H2B-GFP expression cassette controlled by a tetracycline-responsive regulatory element (TRE-H2B-GFP)(Figure 1A). Upon doxycycline administration in the drinking water (pulse), compound villin-rtTA/TRE-H2B-GFP animals show complete labeling of the intestinal epithelium (Figure 1B–C) [27]. Following doxycycline withdrawal (chase), expression of H2B-GFP fusion protein is silenced (Figure 1D) and, due to the high intestinal turnover rate, labeled cells are progressively cleared (Figure 1E–I). Label-retaining cells (LRCs) are retained within the lower third of the small intestinal crypt for at least 79 days (Figure 1I and Figure S1).

### LRCs Localize at the Crypt Base and do Not Express Markers of Intestinal Proliferation and of Goblet and Enteroendocrine Differentiation

**Figure 2:**
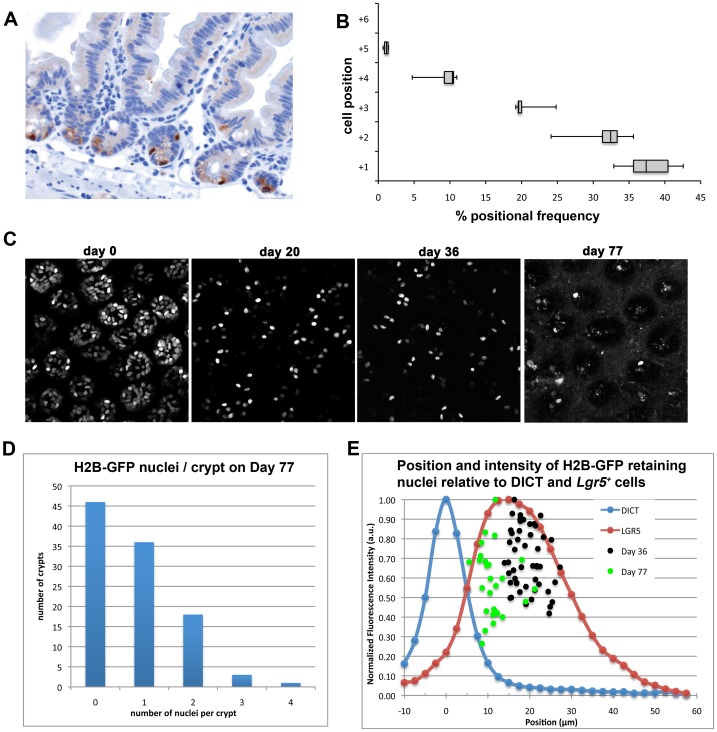
Mapping of Label Retaining Cells along the crypt-villus axis. Mapping of LRCs was performed in small intestinal samples stained for GFP by IHC (A, brown) from animals chased for 4–6 weeks. (B) Box and whisker plots of the frequency at which LRCs were positioned relative to the bottom of the small intestinal crypt (+1). A total of 594 LRCs were mapped to a given position. The majority (90.2%) localize to position +1 to +3. The remaining 9.8% of LRCs appear to map to position +4 and +5. (C–E) Analysis of H2B-GFP retention by means of multiphoton microscopy. (C) Representative images of H2B-GFP expression of intestinal crypts at days 20, 36, and 77 following doxycycline withdrawal on day 0. Images are single planes selected near the base of the crypt from within Z-stacks having X–Y–Z dimensions of 250×250×60 µm. Note that different contrast adjustments have been made for each image, and that intensities are therefore not directly comparable between images. (D) Histogram based on three dimensional analysis showing the number of crypts containing 0, 1, 2, 3, or 4 nuclei at day 77 following removal from doxycycline. 85 Nuclei were counted in 104 crypts, and the average number of cells per crypt is 0.82±0.9. Note that while many crypts show no H2B-GFP positive nuclei, other crypts have multiple H2B-GFP nuclei. (E) Position and intensity of H2B-GFP retaining nuclei relative to the DICT layer at the crypt base and LGR5. DICT was imaged using collagen SHG. The X-axis of the graph (which corresponds to the Z axis of the data volumes) has been set so that ‘0’ coincides with the center of the DICT peak. The average separation between the centroids of the DICT and LGR5 peaks was 17.6±5.02 µm. The average separation between the centroids of the DICT peak and the average position of the H2B nuclei was 19.4±3.5 µm for short (n = 49 nuclei) and long 11.30±3.46 µm (n = 27 nuclei) chases, which indicates that over time the label retaining nuclei occupied positions closer to the crypt base. There is no apparent relationship between nuclear position and H2B-GFP intensity on either day 36 or day 77. Nuclear intensities have been normalized against the brightest nucleus measured on each day, and are therefore not comparable between days on this graph.

To investigate the position and frequency of the LRCs in more detail, we first employed IHC analysis of cross-sections of chased mice to show that H2B-GFP LRCs cluster between positions +1 and +5 from the base of the crypt (Figure 2A,B). Next, we visualized the intestines of pulse-chased animals in 3D by means of multiphoton microscopy (Movies S1 and S2). This approach enabled the identification and localization of LRCs within the entire volume of the lower third of the crypt, rather than looking at cross-sections (Figure 2C). Intestines were analyzed from doxycycline-treated mice, after a medium- (20 or 35 days) and a long-term chase (77 days). In agreement with our studies by IF and on histological cross-sections by IHC, we found an average of 7.04±2.63 LRCs per crypt after 20 days of chase (data not shown). In mice chased for over 70 days, LRCs were still present at an average of approx. 1 per crypt (0.875±0.8)(Figure 2D). To determine the relative position of LRCs along the crypt-villus axis, we measured their distance from the dense irregular layer of connective tissue (DICT) located at the crypt base. The collagen in the DICT layer generates a strong signal when examined using Second Harmonic Generation (SHG) and thus acts as a robust standard to measure the distance of individual LRCs from the base of the crypt. The average position of LRCs was 19.4+/−3.5 µm and 11.3+/−3.5 µm to the DICT at 36 and 77 days of chase, respectively (Figure 2E). The observed downward-shift of LRCs with increasing chase time is most likely due to the fact that more terminally differentiated Paneth cells retain the H2B-GFP label for longer times and are located at lower positions. As a reference, *Lgr5* ^+^ cells in *Lgr5*-EGFP-IRES-CreER^T2^ knock-in mice (here referred to as *Lgr5*-EGFP) [14] are located at 17.6 µm +/5.02 µm from the DICT (peak to peak)(Figure 2E).

Taken together, the data show that the LRCs are located at the base of the crypt at a position largely overlapping with that of *Lgr5* ^+^ cells.

**Figure 3:**
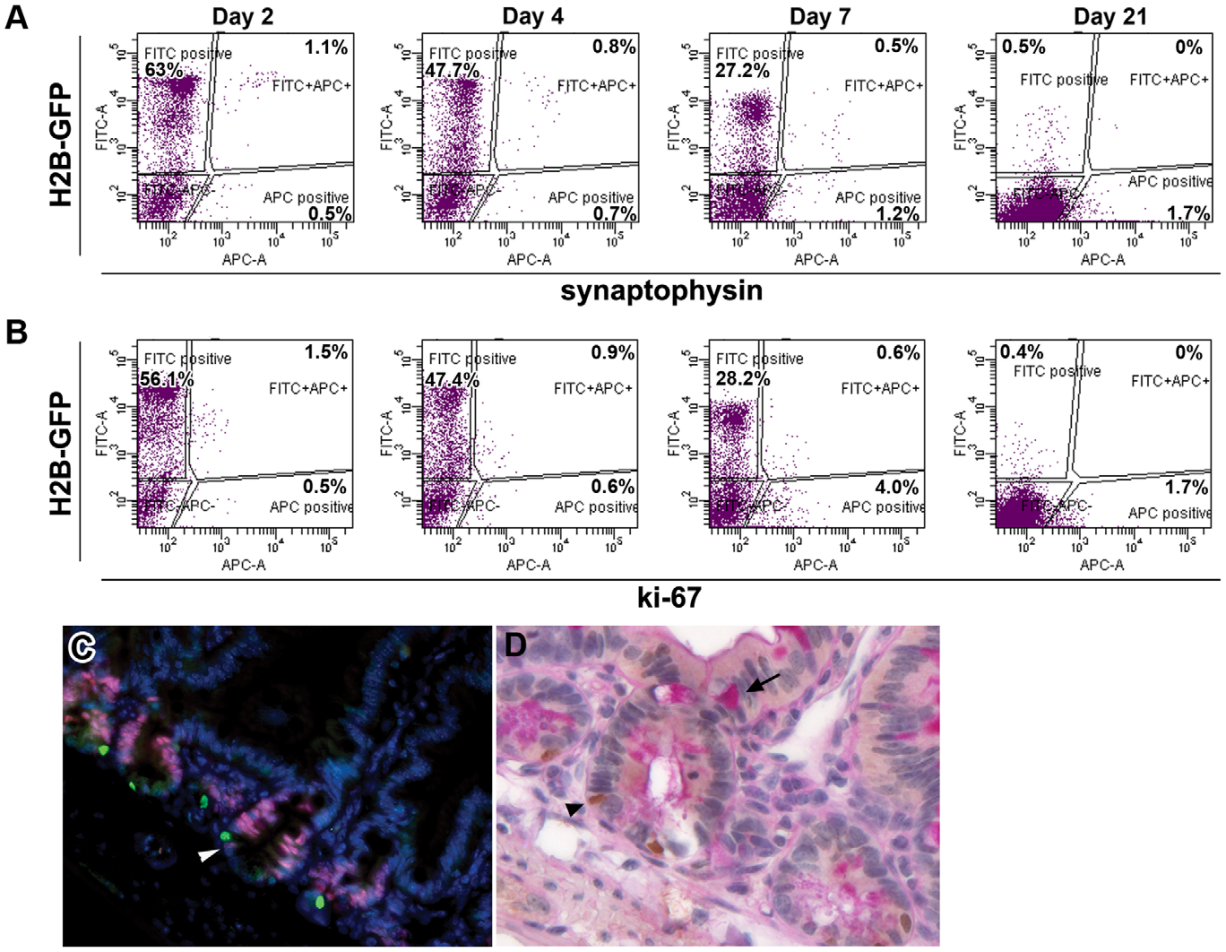
Analysis of proliferation and differentiation markers in LRCs. (A–B) Progressive loss of expression of differentiation and proliferation markers in LRCs following doxycycline withdrawal. The enteroendocrine differentiation marker synaptophysin (A) and the proliferation marker Ki-67 (B) were detected by APC-A whereas FITC-A detects H2B-GFP. The size of each population is indicated as the percentage of epithelial cells. (C–D) Analysis of small intestinal sections from villin-rtTA/TRE-H2B-GFP mice chased for 5 weeks. (C) LRCs (GFP, green) and cycling cells (ki-67, red), the white arrowhead points to a cycling crypt base columnar cell (ki-67, red); (D) LRCs (GFP, arrowheads, brown) and goblet cell marker PAS (arrows, red).

Intestinal quiescent stem cells are expected not to express differentiation and proliferation markers. Accordingly, we analyzed the newly identified LRCs by FACS at different chase times to assess co-expression of H2B-GFP with the enteroendocrine differentiation marker synaptophysin (Figure 3A) and with the proliferation marker Ki-67 (Figure 3B). As shown in Figure 3A–B, both markers are progressively lost from the H2B-GFP^+^ population within 21 days. The resting (G_0_) state of LRCs was confirmed by the lack of Ki-67 staining in GFP^+^ cells (Figure 3C). Of note, cycling crypt base columnar cells were labeled by Ki-67 staining (Figure 3C, arrowhead). Likewise, co-staining with antibodies directed against GFP together with PAS (Periodic-Acid-Schiff) staining confirmed that LRCs do not express this goblet cell-specific differentiation marker (Figure 3D).

### LRCs Co-express Paneth and Stem Cell Markers

**Figure 4:**
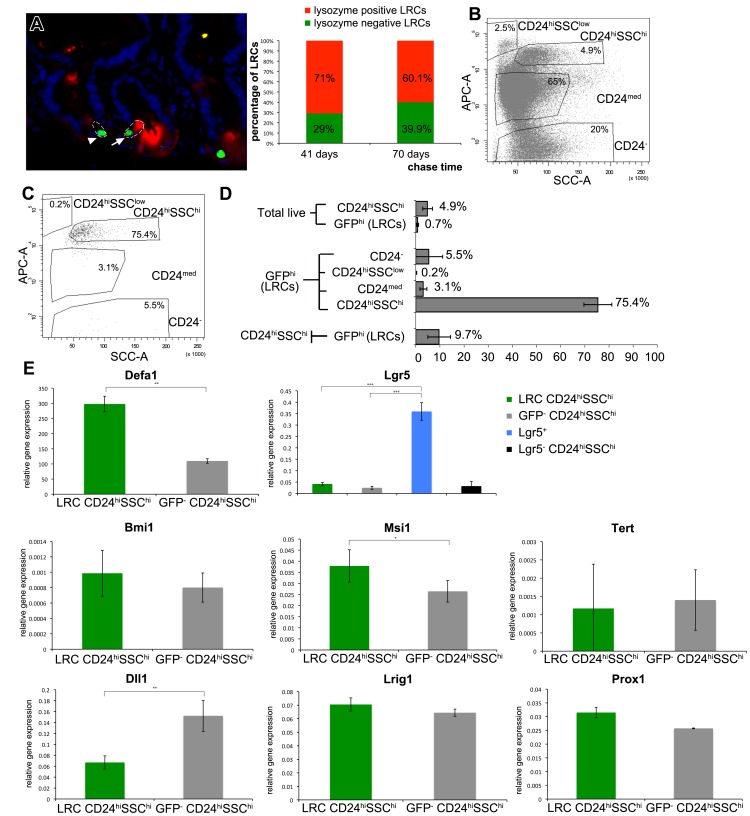
Characterization of LRCs for Paneth and stem cell markers. (A) Co-staining of small intestinal sections from villin-rtTA/TRE-H2B-GFP mice chased for 79 days of LRCs (arrow and arrowhead, GFP, green) and Paneth cells (lysozyme; red). Several (arrow) but not all (arrowhead) LRCs are positive for the Paneth cell marker lysozyme. Imaginary cell boundaries have been drawn (white dotted line). The number of lysozyme-positive and lysozyme-negative LRCs was quantified at chase day 41 and 70. Between 29% (chase 41 days) and 39.9% of LRCs are not expressing lysozyme. (B) Representative FACS image of mouse small intestinal epithelial crypt cells analyzed for the Paneth cell marker CD24. Paneth cells fall commonly within the CD24^hi^SSC^hi^ gate. The size of each population is indicated as percentage of live epithelial cells. (C) Representative FACS image of label retaining cells (chase 75 days) analyzed for the Paneth cell marker CD24. The size of each population is indicated as percentage of live epithelial cells. The majority of LRCs falls directly into the CD24^hi^SSC^hi^ gate, while others scatter just around the CD24^hi^SSC^hi^ gate in close proximity. Some of these cells in close proximity to the CD24^hi^SSC^hi^ gate fall within the upper edge of the CD24^med^ gate. Of note, only a very small minority of LRCs falls outside of this cell cluster and is CD24^-^ (5.54%). (D) Composition of LRCs according to CD24. Displayed are averages of five independent mice chase for 72 to 78 days (± s.d.). The percentage of CD24 subpopulations within the LRCs does not add up to 100%, as some cells fall outside of the drawn gates. (E) Plots of relative gene expression levels (qRT-PCR) of Defa1, Lgr5, Bmi1, Msi1, Tert, Dll1, Lrig1, and Prox1. CD24^hi^SSC^hi^ LRCs (dark green) and their H2B-GFP-negative counterpart (grey) were sorted from five different animals and analyzed by qRT-PCR. Comparison of the marker expression at the two different chase time points (64 days and 83 days) did not show any significant differences (data not shown). Therefore, data from all five animals were analyzed together and shown here. Displayed are averaged β-actin normalized values (± s.e.m.) and the corresponding p-values obtained by two-sample t-test (* <0.02; ** <0.005; *** <2x10e-6). Analysis of Lgr5 was also performed in two populations sorted from 3 Lgr5-EGFP mice, namely Lgr5^+^ (blue) cells and Lgr5^-^CD24^hi^SSC^hi^ (black) cells, to compare the Lgr5 expression levels in Lgr5-EGFP cells (blue) with that of CD24^hi^SSC^hi^ LRCs (dark green).

Paneth cells are long-lived, terminally differentiated cells and thus potentially label-retaining [28], [29]. The average life span of Paneth cells has been estimated around 60 days [29]. In the duodenum, we were able to detect LRCs for up to 100 days of chase (data not shown). These cells were located within the Paneth cell zone of the crypt and often (Figure 4A, arrow and red bar), though not exclusively (Figure 4A, arrowhead and green bar), stained positively for the Paneth cell marker lysozyme.

To further clarify the relationship between LRCs and Paneth cells, we applied the recently reported CD24^hi^SSC^hi^ FACS gating strategy (Figure 4B) [30]. CD24/SSC FACS analysis of crypt epithelial cells from compound villin-rtTA/TRE-H2B-GFP animals chased for longer than 70 days revealed that LRCs represent about 0.7% of the small intestinal crypt cells. The majority of LRCs falls directly into the CD24^hi^SSC^hi^ gate (75.4%, Figure 4C,D), where lysozyme-positive cells were previously reported to cluster [30]. In fact, the vast majority, if not all, of the LRCs seem to represent a homogeneous cell population as those cells falling outside the CD24^hi^SSC^hi^ gate are scattered around it (Figure 4C). Some of the cells located in close proximity to the CD24^hi^SSC^hi^ gate fall within the upper edge of the CD24^med^ gate (Figure 4C,D; 3.1% CD24^med^). Of note, only a very small minority of LRCs does not express CD24 and clearly falls outside of this cluster (Figure 4D; 5.5% CD24-negative).

Next, we isolated the different CD24^hi^SSC^hi^ H2B-GFP^+^ (LRCs) and H2B-GFP^-^ (non label-retaining) subpopulations to perform quantitative RT-PCR (qPCR) expression analysis of genes known to earmark Paneth cells (*Defa1*) as well as cycling and quiescent stem cells (*Bmi1* [18], *Lgr5* [14], *Msi1* [31], *Prox1* [32], *Tert* [19], *Dll1* [33], *Lrig1* [34]). As a control for the qPCR analysis, we employed *Lgr5*-EGFP knock-in mice which express EGFP under the control of the endogenous *Lgr5* gene promoter [14]. qPCR analysis of H2B-GFP^+^CD24^hi^SSC^hi^ cells (LRCs) and their GFP-negative counterpart was performed from five villin-rtTA/TRE-H2B-GFP animals chased for 64 (2 mice) and 83 days (3 mice), respectively. None of the markers showed significant expression differences between samples taken at 64 and 83 days of chase (not shown). Consequently, data from all five animals were combined and displayed in Figure 4E. High *Defa1* expression confirmed the Paneth cell identity of H2B-GFP^+^CD24^hi^SSC^hi^ LRCs. Of note, LRCs within the CD24^hi^SSC^hi^ gate show significantly higher *Defa1* expression than their GFP-negative counterpart (p<0.005). Expression of *Lgr5* was very low in LRCs (when compared with *Lgr5* ^+^ cells) and did not significantly differ between CD24^hi^SSC^hi^ LRCs and H2B-GFP^-^CD24^hi^SSC^hi^ cells. The latter was also true for *Bmi1*, *Tert*, *Lrig1*, and *Prox1*. Notably, *Dll1* expression was significantly lower in CD24^hi^SSC^hi^ LRCs compared to H2B-GFP^-^CD24^hi^SSC^hi^ cells (p<0.005). Among the stem cell markers, only *Msi1* was significantly higher expressed in LRCs (p<0.02).

To obtain further confirmation of the Paneth identity of LRCs but also to better characterize their gene expression patterns, we obtained genome-wide transcriptional profiles from H2B-GFP^+^ and H2B-GFP^-^ intestinal cells sorted by FACS after at 46–47 days of chase (Table S1). Several Paneth-cell specific genes including *Mmp7* (37×), *Defa4* (20×), *Cd24* (14×), *Lyz* (lysozyme; 11×), *Defcr5* (11×), *Sox9* (10×), and *Defb1* (9×) are upregulated among H2B-GFP^+^ cells (Table S2). Also, comparison of these profiling data with those obtained by Sato et al. by comparing CD24^hi^SSC^hi^ cells with *Lgr5^+^* CBCs [30], revealed a large number of common genes thus confirming that the majority of LRCs fall within the CD24^hi^SSC^hi^ Paneth-specific FACS gate (Table S3).

Overall, these results indicate that label-retaining cells have a lifespan of up to 100 days and show a Paneth-like cell identity as indicated by *Defa1* expression and by their CD24^hi^SSC^hi^ FACS phenotype. Furthermore, LRCs express relatively high levels of the stem cell marker *Msi1*.

### Intestinal LRCs: Stem or Niche Cells?

**Figure 5:**
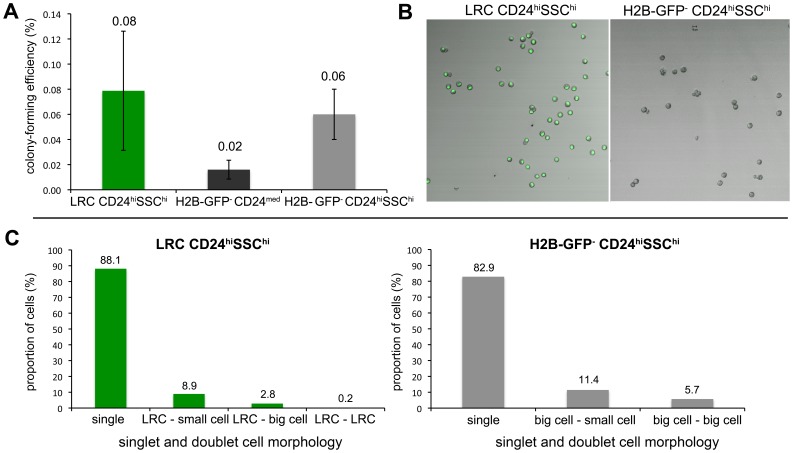
Organoid-formation assay. (A) Organoid-forming capacity of sorted populations from villin-rtTA/TRE-H2B-GFP mice, chased for 72–78 days. Displayed are average percentages of organoids formed (±s.e.m.) from 5000 FACS-sorted and plated events in 5 independent experiments as scored 14 days after plating. (B–C) To assess the presence of doublets among the sorted LRCs, the different subpopulations were examined by confocal microscopy. 500 cells were sorted in each well of a 6-well plate, imaged by confocal microscopy (B) and counted (C). (B) Representative IF images showing the composition of the LRC CD24^hi^SSC^hi^ population (left) and the H2B-GFP^-^ CD24^hi^SSC^hi^ population (right). (C) Quantification of the composition of FACS-sorted LRC CD24^hi^SSC^hi^ (left) and H2B-GFP^-^ CD24^hi^SSC^hi^ (right) by morphology for single cells and aggregates of differently sized cells; displayed are percentages.

The observed *Msi1* expression in LRCs indicates that, next to their Paneth cell identity, they may be multipotent, i.e. capable of giving rise to the various differentiated epithelial lineages of the adult GI tract, even in homeostasis. To test this hypothesis we applied previously established culture conditions [35] to generate *ex vivo* crypt-villus organoids from LRCs thus assaying their multipotency in comparison with *Lgr5* ^+^ stem cells. To this aim, we employed CD24/SSC FACS to sort H2B-GFP^+^ and H2B-GFP^-^ cells from pulsed-chased villin-rtTA/TRE-H2B-GFP animals and, as control, *Lgr5*-GFP^+^ cells from *Lgr5*-EGFP mice [14]. Figure 5A shows the average percentages of organoids formed in five independent experiments carried out with FACSorted single cells obtained from small intestinal crypts of pulsed-chased villin-rtTA/TRE-H2B-GFP animals (chase time 72–78 days). Organoids were obtained from LRCs (H2B-GFP^+^CD24^hi^SSC^hi^) at higher frequencies (∼4 fold, 0.08%) than from H2B-GFP^-^CD24^med^ cells (0.02%), i.e. the sorting gate where *Lgr5* ^+^ cells are known to reside (Figure 5A and S7I) [30]. Their GFP-negative counterpart (H2B-GFP^-^CD24^hi^SSC^hi^) was able to form organoids with a frequency (0.06%) comparable to that of LRCs (Figure 5A). Of note, CD24-negative LRCs, which constitute 5.5% of all LRCs (Figure 4D), were not able to give rise to organoids (data not shown). LRCs-derived organoids were morphologically indistinguishable from those obtained from GFP-negative cells and from whole crypts, and encompassed all differentiated cell types, namely enterocytes, Paneth, goblet, and enteroendocrine cells. Also, *Lgr5* expression was detected by RT-PCR in the LRC-derived organoids (Figure S2).

As recently reported, doublets of *Lgr5* ^+^ (cycling stem cells) and CD24^hi^SSC^hi^ (allegedly Paneth) cells have increased organoid-forming capacity when compared with single *Lgr5* ^+^ cells (up to 80 fold) [30]. We performed similar control experiments by employing *Lgr5*-EGFP mice and tested the organoid-forming capacity of different sub-populations FACSorted according to their *Lgr5*-GFP staining (here indicative of *Lgr5* expression) and to the CD24^hi^SSC^hi^ markers (Figure S3A). While *Lgr5* ^+^ single cells showed a very low organoid-forming capacity, Lgr5-GFP^+^CD24^hi^SSC^hi^ doublets (i.e. composed of *Lgr5* ^+^ and Paneth cells) were significantly (∼70 fold) more efficient in forming viable organoids. The composition and percentages of single cells and aggregates within each population is depicted in Figure S3B.

Because of their size, these *Lgr5* ^+^CD24^hi^SSC^hi^ doublets are likely to contaminate the CD24^hi^SSC^hi^ sorting gate in the LRC-sorting experiments even when stringent single cell selection is applied. Highly stringent gating for single cells was performed in a two-step strategy and is explained in detail in the Methods Section (Figure S7). To assess the presence of doublets among the sorted LRCs, we examined the different subpopulations by confocal microscopy (Figure 5B). To this aim, 500 cells were sorted in each well of a 6-well plate, imaged by confocal microscopy and counted. Figure 5B shows representative images of each H2B-GFP^+^CD24^hi^SSC^hi^ (left) and H2B-GFP^-^CD24^hi^SSC^hi^ population (right). The percentage of single cells and doublets present in sorted CD24^hi^SSC^hi^ LRCs (Figure 5C, left, 8.9%) and in H2B-GFP^-^CD24^hi^SSC^hi^ (Figure 5C, right, 11.4%) cells is displayed. However, *Lgr5* expression was found to be very low in the sorted LRCs (Figure 4E) and did not differ between CD24^hi^SSC^hi^ LRCs and H2B-GFP^-^CD24^hi^SSC^hi^ cells. Hence, although the “contaminating” presence of *Lgr5*-Paneth doublets in the CD24^hi^SSC^hi^ FACS gate do not seem to represent a problem for the expression analysis, their organoid-formation capacity is likely to be of a much larger order of magnitude and does not allow us to draw any definitive conclusion on the capacity of single LRCs to form organoids.

In confirmation of a previously published study [30], these data point to an essential role of Paneth-like CD24^hi^SSC^hi^ cells in providing niche support to actively cycling *Lgr5* ^+^ cells during homeostasis.

### Label-retaining Cells Respond to Radiation-induced Tissue Injury by Entering the Cell Cycle and Acquiring Stem-like Characteristics and by Losing their Paneth Cell Identity

**Figure 6:**
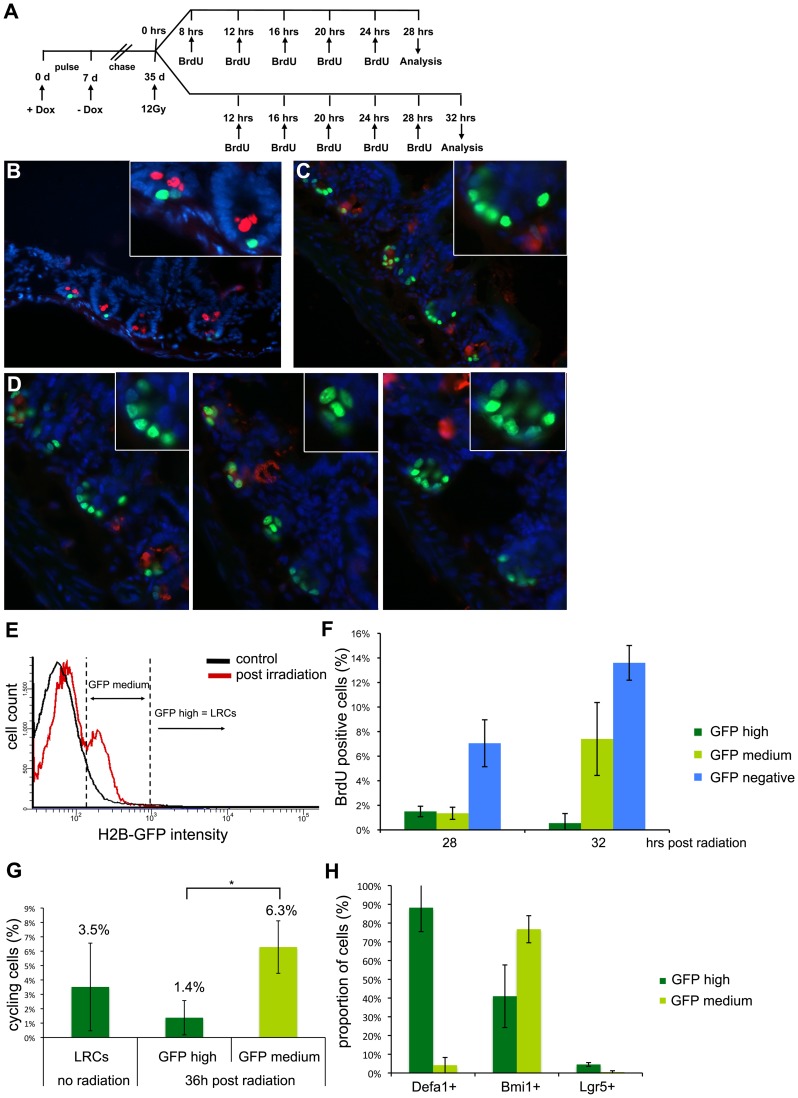
Proliferative response of LRCs upon radiation-induced tissue injury. (A) Experimental scheme. (b–d) IF analysis for GFP (green, LRCs) and lysozyme (red, Paneth cells) relative to a non-irradiated control animal at four weeks of chase (B) and to two irradiated animals that were treated according to the scheme in (A) analyzed at 32 hours after irradiation (C,D). (E) Overlay of representative image of FACS analysis of intestinal epithelial cells of non-irradiated animals at four weeks of chase (black) compared to animals at 32 hours after whole-body irradiation (red, treated according to the scheme in 6A) for H2B-GFP intensity. Two GFP-positive populations, GFP^hi^ and GFP^med^ are resolved. Following radiation-induced tissue injury, a new GFP^med^ population appears (red peak), which is almost absent in untreated control animals (black line). (F) BrdU uptake as a measure of the LRCs’ proliferative response to tissue injury. The GFP^hi^ and GFP^med^ populations were evaluated for the percentage of BrdU^+^ cells at 28 and 32 hrs following radiation as depicted in A. The average BrdU levels (±s.d.) measured in 2 independent mice are displayed. (G) CyclinD1-expression as a measure of the LRCs’ proliferative response to tissue injury. Displayed is the percentage of CyclinD1-expressing cells from all Actb-positive ones as determined by single-cell RT-PCR. LRCs were obtained from three pulse chased animals; GFP^hi^ and GFP^med^ cells were obtained from three pulse-chased animals and isolated 36 hrs. after irradiation. Single cell RT-PCR was carried out on a total of 3×90 LRCs, 3×60 GFP^hi^ and 3×120 GFP^med^ cells. Single cells were only included in the analysis when the housekeeping gene Actb was expressed. The average percentages of CyclinD1-expressing cells from all Actb-positive ones (±s.d.) and the corresponding p-values obtained by two-sample t-test (* <0.02) are shown. (h) Percentage of GFP^hi^ and GFP^med^ cells expressing Defa1, Bmi1, and Lgr5 as determined by single cell RT-PCR. FACSorted GFP^hi^ and GFP^med^ cells were isolated 36 hrs. after radiation-induced tissue injury. Single-cell RT-PCR was carried out on a total of 2×30 GFP^hi^ and 2×60 GFP^med^ cells from each one male and one female mouse. Gene expression status of single cells was only included in the charts when the housekeeping gene Actb was expressed. The average proportion of cells expressing each gene (±s.d.) is displayed.

Quiescent stem cells are thought to play a rate-limiting role in the regenerative process following tissue injury as previously shown in other stem cell niches [3], [5], [6], [10]. In the mouse GI tract, tissue injury can be experimentally simulated by whole-body exposure to ionizing radiation leading to complete cell death of crypt cells (crypt clearance). Irradiated crypts progressively shrink and eventually disappear within two days [36]. After a lag in growth following a single 12 Gy radiation dose, new outgrowing crypts are thought to result from single clonogenic cells [36]. To study the behavior of LRCs upon tissue injury, villin-rtTA/TRE-H2B-GFP mice were exposed to a single 12 Gy dose following a four-week chase period. After an 8–12 hour recovery time, animals were injected five times with BrdU at 4-hour intervals to mark proliferating cells. Animals were analyzed 28 and 32 hours after irradiation, respectively (Figure 6A). When compared with non-irradiated controls (Figure 6B), several clusters of multiple H2B-GFP positive cells were observed in the small intestines of mice analyzed at 32 hours after radiation (Figure 6C,D). These results are indicative of the proliferative activation of LRCs upon tissue injury, as also suggested by the observed diminished H2B-GFP intensity within the LRC clusters due to the progressive dilution of the H2B-GFP signal after each cell division cycle.

In order to quantify the levels of H2B-GFP at this time point, FACS analysis of live epithelial cells was applied 32 hrs. after radiation: a distinct population of cells with intermediate GFP intensity (GFP^med^) when compared with the parental LRCs (mainly GFP^hi^) becomes evident upon irradiation (Figure 6E). To ensure that the observed GFP-levels do not result from non-specific, endogenous fluorescence or radiation-induced expression of the H2B-GFP transgene, sham-pulsed mice were subjected to the same treatment (Figure S4A). Sham-pulsed animals and doxycycline-pulsed and chased mice were analyzed at 32 hours after radiation (Figure S4A), the same time point when the above-mentioned cell clusters (Figure 6C,D) and the GFP^med^ population (Figure 6E) appeared. GFP-levels of sham-pulsed animals, when compared to littermates treated with doxycycline and chased, were close to background levels (Figure S4B). The GFP^hi^ population was absent in sham-pulsed mice and a significantly smaller fraction of GFP^med^ cells was measured (Figure S4B, p<0.001). Hence, irradiation and BrdU-injection by itself does not induce the expression of the H2B-GFP transgene. On the contrary, the GFP^med^ cells detected after radiation descend from the population of cells that retained the H2B-GFP-protein prior to the radiation insult.

To confirm that the GFP^med^ population results from cell cycle activation of H2B-GFP LRCs, BrdU-uptake was compared between GFP^med^ and GFP^hi^ cells at 28 and 32 hours after irradiation (Figure 6F). While at 28 hrs. after radiation, the proliferation rates of GFP^med^ and GFP^hi^ cells did not differ, at 32 hrs., i.e. the time point when clusters of H2B-GFP positive cells were observed (Figure 6C,D), GFP^med^ cells show a clear increase in BrdU-uptake when compared to GFP^hi^ cells (Figure 6F). Hence, the GFP^med^ cells descend from H2B-GFP LRCs which react to the tissue insult by entering the cell cycle thus incorporating BrdU and progressively diluting the GFP-labeled chromatin. The proliferative response of LRCs to tissue injury was confirmed by single-cell RT-PCR (Figure 6G). Only a small proportion of the quiescent cells (i.e. LRCs from non-irradiated mice) and of the GFP^hi^ cells following irradiation expressed CyclinD1. In contrast, the percentage of CyclinD1-expressing cells in the GFP^med^ population was significantly higher than in the GFP^hi^ cells upon tissue insult (Figure 6G, p<0.02).

The presence of clusters of H2B-GFP nuclei in each crypt and the decrease of H2B-GFP intensity following radiation-induced tissue injury was confirmed and quantified by multiphoton microscopy (Figure S5). Here, we examined the number and intensity of LRCs after a short-term chase (35 days). Consistent with a stimulation of proliferation following tissue injury, there was a significantly increased number of H2B-GFP^+^ cells at 3 and 5 days following gamma irradiation (12 Gy)(Figure S5A) with altered position (Figure S5B) and a significantly reduced GFP intensity (Figure S5C).

To further characterize the GFP^hi^ and GFP^med^ cells arising upon irradiation, single-cell RT-PCR analysis was carried out for the *Defa1*, *Bmi1* and *Lgr5* genes (Figure 6H). As predicted by the previously reported radio-resistance of Paneth cells [28], the GFP^hi^ fraction encompasses a majority of *Defa1*-expressing cells when compared with GFP^med^ cells. Instead, the GFP^med^ population is clearly enriched in *Bmi1* ^+^ cells (Figure 6H). Notably, only very few GFP^med^ cells expressed *Lgr5* (Figure 6H), thus suggesting that *Bmi1* ^+^ cells but not crypt base columnar cells are direct descendants of LRCs upon radiation-induced tissue injury. Complete regeneration following the radiation dosage of 12 Gy is very efficient and is completed within the first four days (Figure S6).

Overall, these results indicate that, upon radiation-induced tissue injury, dormant LRCs exit the G_0_ phase of the cell cycle and start dividing as part of the physiologic regenerative response of the intestinal epithelium. During this process, LRCs loose markers of Paneth cell differentiation acquire *Bmi1* expression and therewith a more stem-like identity.

## Discussion

The existence within the crypts of Lieberkühn of dormant cells which become activated and proliferate in response to tissue insults is reminiscent of the scenario observed in other stem cell niches, e.g. the stomach antrum [7], [8] and the bone marrow [9], [10], [37], where, while resident stem cells are responsible for the daily homeostasis, dormant stem cells are activated and recruited upon tissue damage [1]. In the mouse GI tract, depletion of cycling intestinal stem cells has first been achieved through the targeted deletion of the *Ascl2* gene whose expression largely overlaps that of *Lgr5* [15]. Notwithstanding the high efficiency of this targeted depletion, complete regeneration of the intestinal epithelium was observed between 6 and 10 days after disappearance of the cycling stem cell population [15]. More recently, an even more complete ablation of *Lgr5*-expressing cells showed that these stem cells are dispensable and that their loss is compensated by the expansion of the *Bmi1^+^* stem cell pool [16]. Upon their activation, *Bmi1^+^* stem cells give rise to *Lgr5^+^* cells together with all other differentiated lineages of the mouse small intestine [16]. Lineage tracing experiments have confirmed that the *Bmi1*-expressing cells at position +4 and the more cycling *Lgr5^+^* CBCs represent stem cell pools with distinct functions in homeostasis and tissue injury, albeit with a bidirectional lineage relationship [16], [24]. More recently, the existence of additional populations of slow-cycling intestinal stem cells capable of underlying the regenerative response upon tissue insults have been demonstrated in several high-impact and elegant publications: apart from *Bmi1* ^+^, also *mTert* ^+^, *Hopx* ^+^ and *Lrig1* ^+^ cells were shown by lineage tracing to represent *bona fide* stem cells capable of compensating loss of actively cycling CBCs and giving rise to *Lgr5* ^+^ cells and to all differentiated intestinal cell types in situations of tissue injury [16], [19], [25], [26].

Tissue injury in the intestine is often due to inflammation and/or dietary mutagens. Experimentally, tissue injury can be simulated by whole-body exposure to ionizing radiation leading to complete sterilization of the epithelial lining followed by tissue regeneration from clonogenic stem cells [36]. Irradiation of *Lgr5*-EGFP mice was shown to result in the depletion of *Lgr5^+^* stem cells followed by a proliferative, regenerative phase [17]. In view the results presented here, the activation and recruitment of Paneth cells capable of de-differentiation into *Bmi1^+^* stem cells is likely to represent yet another mechanism to account for the rapid regenerative process upon loss of *Lgr5* ^+^ cycling stem cells following radiation-induced tissue injury. Our results are indicative of an entirely novel functional role for the infrequently dividing, Paneth cells as source of stem cells upon tissue injury. When massive cell death is induced by irradiation, Paneth/LRCs survive because of their intrinsic radio-resistance [28] and become activated to proliferate and acquire a stem-like identity, as shown by *Bmi1* expression [16], [18] and the simultaneous loss of their Paneth cell identity. Nevertheless, given the increasing complexity of subpopulations of slow-cycling stem cells capable of a regenerative response upon (radiation-induced) tissue injury, their hierarchical relationship remains unclear. The Paneth/LRCs here identified do not express *mTert* and *Lrig1* during homeostasis. Future experiments will have to address whether *Tert*- and *Lrig1*-expressing cells descend (directly or through the *Bmi1* ^+^ cells) from Paneth/LRCs following tissue injury or if they represent a distinct population of quiescent stem cells.

Although Paneth cells play an essential role both as integral part of the *Lgr5* niche [30] and during the regenerative response (this study), their functional relevance has been challenged by the generation of mouse strains where this lineage is ablated to different extents: *Gfi1* ^−/−^ [38], a conditional deletion of *Sox9* [39], [40], a transgenic model where diphtheria toxin A expression is controlled by the Paneth cell-specific cryptdin 2 (*Cr2*) promoter [41], and, more recently, a conditional Atoh1^−/−^ model [42]. As none of these models show a morbid phenotype, doubts have been raised relative to the functional relevance of Paneth cells in the upper intestinal tract. However, with the only possible exception of the study by Kim et al. [42], Paneth cell numbers are reduced among these mice though not entirely depleted [43], [44], which does not allow any definitive conclusion relative to Paneth cells’ functional relevance. Moreover, the multiplicity of *Lgr5^+^* stem cells was shown to be reduced among the majority these models [30] though not in the *Atoh1*-deficient mice [42]. More importantly, in the above studies the Paneth cell depleted mouse models were never challenged by chronic inflammation or irradiation to assess their regenerative capacity when compared with wild type mice. In fact, the adaptive response to small bowel resection is subtly impaired in *Atoh1* ^−/−^ crypts compared to *Atoh1*-proficient crypts in the same mosaic mice [45]. Also, increased apoptosis after DSS-induced injury was observed in *Atoh1* ^−/−^ crypts (Dr. N. Shroyer, personal communications).

Paneth cells are found in the small intestine yet not in the colon where otherwise uncharacterized CD24^+^ cells are intermingled with *Lgr5* ^+^ stem cells similar to the organization of Paneth and *Lgr5* ^+^ cells in the upper intestine [30]. More recently, CD24^+^/c-Kit^+^/CD117^+^ secretory cells have been characterized in the mouse colon which support Lgr5^+^ stem cells [46]. Notably, Paneth cell metaplasia, i.e. the unusual appearance of Paneth cells in the colon, has been reported in patients affected by chronic inflammatory bowel diseases such as Ulcerative Colitis [47], [48]. Also, genetic analysis of the role played by Wnt/§-catenin signaling in Paneth cell differentiation has shown that expression of markers such as *Lyz*, *Pla2g2a* and several defensins immediately follows loss of *Apc* function in both the small and large intestine of the mouse [49]. Likewise, early sporadic human adenomas reveal expression of the same markers of Paneth cell differentiation [49]. In fact, Paneth cell metaplasia is not only observed in colorectal adenomas but also in the non-neoplastic mucosa surrounding the adenomatous lesion often carrying early genetic alterations (e.g. *KRAS*) [50]. A second study on a mouse model of dietary-induced sporadic colon cancer showed that the very first measurable change before tumor onset consists of ectopic expression of Paneth cell markers together with transcriptional activation of *Fzd5* and *EphB2*, two members of the Wnt signaling cascade necessary for Paneth cell differentiation and localization [51].

In view of our results, Paneth cells metaplasia in colorectal adenomas together with the observed early expression of Paneth cell markers upon constitutive Wnt signaling activation in preneoplastic colonic epithelium are highly suggestive of a functional role of Paneth cells in inflammation and colorectal tumor initiation. On one hand, metaplastic Paneth cells may support the nascent *Lgr5* ^+^ cancer stem cell [52] analogous their role as niche of normal CBCs [30]. On the other hand, upon chronic inflammation, metaplastic Paneth cells may represent source of activated *Bmi1* ^+^ stem cells which, because of their increased proliferation rate, may acquire somatic mutations at oncogenes and tumor suppressors thus underlying cancer onset.

Recently, Sei et al. reported that few enteroendocrine cells earmarked by cholecystokinin-GFP expression localize at the crypt base where the stem cells reside [53]. These cells express several intestinal stem cell markers (*Lgr5*, *Dcamkl1*, *Cd133*) together with multiple endocrine hormones. The results suggest that a subset of enteroendocrine cells migrates down to the crypt base or stays localized at the crypt base, where they express, analogous to the Paneth cells in our study, both stem and postmitotic endocrine markers. In view of these data, rather than a dichotomy of cycling and quiescent stem cells, the intestinal stem cell niche seems to encompass a continuum of cellular phenotypes (e.g. different degrees of proliferation rates, pluripotency and plasticity) with postmitotic, apparently fully differentiated cells capable of de-differentiation and acquisition stem-related functions.

In conclusion, we identified Paneth cells as long-term label retaining cells which, in the presence of tissue insults, switch from a resting to a proliferative, stem-like state and as such actively contribute to the regenerative response of the injured intestinal epithelium.

## Methods

### Ethics Statement

This study has been approved by the stichting Dier Experimenten Commissie (DEC), approval number EUR1383.

### Mice

Transgenic Villin-rtTA2-M2 animals [27] were bred with tetO-HIST1H2BJ/GFP (H2BGFP) animals [3]. Transgene expression was induced in compound heterozygous animals and their littermates by replacing normal drinking water with 5% sucrose water containing 2 mg/ml doxycycline (Sigma, D9891). Dox-treated water was changed every 2 days. After 7 days, doxycycline treatment was stopped and mice received regular drinking water again. At defined time-points after doxycycline-withdrawal, mice were sacrificed and tissues were analyzed for H2B-GFP-expression.

### Radiation and BrdU Injection

Animals were exposed to 12 Gy of whole-body γ-radiation. Mice were injected intraperitoneally at 4-hour intervals with 100 µl BrdU solution in PBS at 10 mg/ml^−1^ (BD Pharmingen).

### Immunofluorescence

GFP (1∶50, A11222, Invitrogen), Ki67 (1∶50, M7249, Dako), BrdU (1∶50, BD Pharmingen) and Lysozyme (1∶1000, A0099, Dako) were employed for Immunofluorescence analysis. Ki67 was detected using rabbit-anti-rat-A594 (Invitrogen), BrdU was detected using goat-anti-mouse-A594 (Invitrogen) and goat-anti-rabbit-A488 (Invitrogen) was used for signal detection of GFP.

### Immunohistochemistry

Tissues were fixed in 4% PFA and embedded in paraffin. Four µm sections were mounted on slides and stained by HE and PAS for routine histology. Immunohistochemistry was performed according to standard procedures using the following antibodies: GFP (1∶800, A11222, Invitrogen), Lysozyme (1∶5000, A0099, Dako), Synaptophysin (1∶750, A0010, Dako), Villin (1∶200, 610359, BD Transductoin Laboratories) and BrdU (1∶250, BD Pharmingen). Signal detection was performed using Rabbit EnVision+ System-HRP (K4011, Dako) for GFP, Lysozyme and Synaptophysin. Villin and BrdU were detected using Mouse EnVision+ System-HRP (K4001, Dako).

### Multiphoton Microscopy

Mice were sacrificed and a ∼1 cm section of the intestine was removed at ∼5 cm distance from the stomach. The intestinal section was rinsed twice in PBS and placed in a glass-bottomed petri dish (Matek) in a few drops of PBS containing the muscle relaxant scopolamine (Sigma) to stop peristalsis and stabilize the sample for imaging. Samples were imaged on a LaVision Biotec TRIM scope using an Olympus 20×, 0.95 NA water immersion objective on an inverted Nikon Eclipse T2000 stand. Samples were illuminated using a Coherent Chameleon II Ultrafast laser at 850 nm. GFP fluorescence and collagen SHG were simultaneously detected using 525/50 and 435/40 nm band-pass filters (respectively).

Images were analysed using Fiji 1.45i (ImageJ) and SigmaPlot. A gaussian curve was fit to the axial profile of SHG intensity in order to determine the centroid and width of the dense irregular connective tissue (DICT) layer at the base of each crypt. The ImageJ macro “3D Objects Counter” was used to determine the intensities and axial positions of the GFP-H2B labeled nuclei within a given crypt. Axial position was then compared to the SHG centroid of the DICT beneath that crypt.

### Crypt Isolation and Cell Culture

Crypts were isolated and cultured according to the protocol kindly provided and previously described by T. Sato, R. Vries, and H. Clevers [35]. Sorted cells were collected in crypt culture medium and embedded in Matrigel containing Jagged-1 peptide (1 mM; AnaSpec), EGF (10–50 ng/ml; Peprotech), R-spondin 1 (1000 ng/ml; R&D Biosystems) and Noggin (100 ng/ml; Peprotech). 500 sorted events were plated per well of a 48-well plate. Crypt culture medium (250 ml for 48-well plates) containing Y-27632 (10 mM) was overlaid. Growth factors were added every other day and the entire medium was changed every 4 days. Organoid counting was done two weeks after plating and percentages were calculated using the total number of sorted events per population.

For passage, organoids were removed from Matrigel and mechanically dissociated into single-crypt domains, and then transferred to fresh Matrigel. Organoids were passaged every 1–2 weeks.

### Cell Preparation for FACS

Small intestinal crypts were isolated as described above. Crypts were spun twice at 600 rpm for 2 min. to remove blood cells and other single cells. Clean crypt preparations were digested into single cells in TrypLE (Invitrogen) for 30 min. at 37°C. The cell suspension was passed through a 40 µm cell strainer, washed by doubling the volume using 2% FCS/PBS, and spun down for 5 min. at 1200 rpm at 4°C. All staining steps were performed for 30 min. at 4°C in the dark. Antibodies: synaptophysin (DakoCytomation) and secondary APC-conjugated F(ab’)2 Donkey-anti-rabbit IgG(H+L) (Jackson); ki-67 (DakoCytomation) and secondary APC-conjugated goat-anti-rat Ig specific (BD Pharmingen); anti-mouse CD24-APC clone M1/69 (BioLegend); APC BrdU Flow Kit (BD Pharmingen); LIVE/DEAD Fixable Dead Cell Stain Kit (L10119, Invitrogen).

### FACS

A detailed description of our gating strategy including representative FACS plots can be found in Figure S7.

**Table 1:** 

Gene	TaqMan assay
actin, beta (*Actb*)	Mm00607939_s1
defensin, alpha 1 (*Defa1*)	Mm02524428_g1
leucine rich repeat containing G protein coupled receptor (*Lgr5*)	Mm00438890_m1
Bmi1 polycomb ring finger oncogene (*Bmi1*)	Mm03053308_g1
Musashi homolog 1(Drosophila) (*Msi1*)	Mm00485224_m1
prospero-related homeobox 1 (*Prox1*)	Mm00435969_m1
leucine-rich repeats and immunoglobulin-like domains 1 (*Lrig1*)	Mm00456116_m1
delta-like 1 (Drosophila) (*Dll1*)	Mm01279269_m1
telomerase reverse transcriptase (*Tert*)	Mm00436931_m1

### Quantitative PCR

CD24^hi^SSC^hi^ LRCs and their GFP-negative counterpart were sorted from crypt preparations of the small intestine of five animals that were chased for 64 (2) and 83 (3) days respectively. Likewise, *Lgr5*-positive cells and CD24^hi^SSC^hi^ were sorted from three different *Lgr5*-EGFP animals. Total RNA was isolated using RNeasy Mini Kit (Quiagen), converted into cDNAs using High Capacity RNA-to-cDNA kit (Applied Biosystems) and employed in a pre-amplification step using the Taqman®PreAmp Master Mix (Applied Biosystems) according to manufacturer’s instructions. The Linearity of pre-amplification was controlled for all primers. Assays were carried out as duplicate reactions using the 7900HT Fast Real Time system (Applied Biosystems). Quantitative PCR was performed for the following markers: *Actb*, *Defa1*, *Lgr5*, *Bmi1*, *Msi1*, *Prox1*, *Lrig1*, *Dll1* and *Tert* using inventoried TaqMan assays (Applied Biosystems, see Table 1) according to manufacturer’s instructions. The Actb gene was used as housekeeping gene. Comparison of the marker expression at the two different chase time points (64 days and 83 days) did not show any differences. Therefore, data from all five animals was analyzed together. Displayed are thus the averages of five animals, the standard error of means and the corresponding p-values obtained by two-sample t-test.

### Single Cell RT-PCR

Single cells were directly sorted into PCR tubes containing 9-µl aliquots of RT-PCR lysis buffer and treated as described previously and two-step multiplex nested single cell RT-PCR was done as described [54]. Multiplex single cell RT-PCR allows the detection of multiple genes expressed in one single cell. RNA-quality control was achieved by using the housekeeping gene *Actb* and examining different gene products at the same time. Primer sequences are displayed in Table 2.

**Table 2:** 

gene	primer sequence
Actb_F1	5′-atatcgctgcgctggtcgtc-3′
Actb_R1_R2	5′-ccggagtccatcacaatgcc-3′
Actb_F2	5′-tggtgggaatgggtcagaag-3′
CyclinD1_F1	5′-gcgtaccctgacaccaatc-3′
CyclinD1_R1	5′-gcttgttctcatccgcctc-3′
CyclinD1_F2	5′-catgcggaaaatcgtggcc-3′
CyclinD1_R2	5′-ggccggatagagttgtcag-3′
Defa1_F1	5′-cctactctttgcccttgtcc-3′
Defa1_R1	5′-ctcatgctcgtcttgttctc-3′
Defa1_F2	5′-agagactaaaactgaggagc-3′
Defa1_R2	5′-caggttccattcatgcgttc-3′
Bmi1 _F1	5′-tgcatcgaacaaccagaatc-3′
Bmi1_ R1	5′-atcattcacctcttccttag-3′
Bmi1 _F2	5′-cactaccataatagaatgtc-3′
Bmi1_ R2	5′-aatctcttcttctcttcatc-3′
Lgr5_F1	5′-acctcagtatgaacaacatc-3′
Lgr5_R1	5′-ggagtccatcaaagcatttc-3′
Lgr5_F2	5′-ttacgtcttgctggaaatgc-3′
Lgr5_R2	5′-gagcattgtcatctagccac-3′

Single cell RT-PCR of *CyclinD1*: LRCs were obtained from three pulse chased animals; GFP^hi^ and GFP^med^ cells were obtained from three pulse-chased animals and isolated 36 h after irradiation. Single cell RT-PCR was carried out on a total of 3×90 LRCs, 3×60 GFP^hi^ and 3×120 GFP^med^ cells. Single cells were only included in the analysis if the housekeeping gene *Actb* was expressed. The average percentage of *CyclinD1*-expressing cells was calculated from the number of *Actb*-positive cells (±s.d.). Single cell RT-PCR expression analysis of the *Defa1*, *Bmi1*, and *Lgr5* genes, performed on FACSorted GFP^hi^ and GFP^med^ cells 36 hrs. following radiation, was carried out on a total of 2×30 GFP^hi^ and 2×60 GFP^med^ cells from each of one male and one female mouse. Single cells were included in the charts only when the housekeeping gene *Actb* was expressed. The average percentage of expressing cells was calculated from the number of *Actb*-positive ones.

### RNA-isolation and RT-PCR

RNA was isolated from organoid cultures using TRIzol Reagent (Invitrogen) and cDNA was synthesized using RevertAid H Minus First Strand cDNA Synthesis Kit (Fermentas) following the manufacturer’s instructions. RT-PCR was done using primers Lgr5_F1 (5′-acctcagtatgaacaacatc-3′) and Lgr5_R1 (5′-ggagtccatcaaagcatttc-3′).

### Microarray Analysis

10.000 H2B-GFP^+^ (LRCs) and H2B-GFP^-^ cells were sorted from the upper intestinal tract of two double transgenic animals at 46 and 47 days of chase. Total RNA was isolated with RNeasy Micro kit (QIAGEN) and quality controlled with RNA 6000 Pico and Nano LabChip kits (Agilent Technologies). Sample labelling, hybridization, staining and scanning were performed on the Affymetrix MOE430 plus2.0 microarray platform using the Two-cycle Labelling kit according to manufacturer’s protocols. Expression levels were extracted from Affymetrix Mouse430_2 arrays and normalized with RMA using Partek® Genomics Suite™ [http://www.partek.com/]. A principle component analysis (PCA) was performed in Partek® and the two groups (LRCs and non LRCs) were analysed using Significance Analysis of Microarrays (SAM, Stanford University) as implemented in Biowisdom’s OmniViz [http://www.biowisdom.com/content/omniviz]. Five-fold difference in expression between the two groups and a false discovery rate below 1% were used as cut-offs. The resulting list of differentially expressed transcripts was uploaded into Ingenuity IPA [Ingenuity Systems, Redwood City, CA, www.ingenuity.com] and BioBase ExPlain system [BIOBASE GmbH, Germany, www.biobase-international.com] as the starting points for the generation of biological networks. Additional annotations were retrieved using the PantherDB [http://www.pantherdb.org/] [55]. In both systems a p-value is calculated determining the probability that each biological function and/or disease assigned to the data set of interest is due to chance alone. Selected genes were visualized in a molecular network using information contained in IPA and show the connectivity of the individual proteins. Additionally, differentially expressed transcripts and genes are classified either by proprietary ontology’s or by MESH terms according to the categories of canonical pathways, therapeutic target, biomarker, and molecular mechanism. The significance of the association between the dataset and the categories is measured by the ratio of the number of proteins from the dataset that map to the category divided by the total number of proteins that map to the canonical pathway.

## Supporting Information

Figure S1 **In vivo identification of infrequently cycling cells (LRCs) from the mouse intestine.** Following doxycycline-withdrawal (chase), expression of H2B-GFP is silenced. Sections of jejunum (A, C, E, G, I) and ileum (B, D, F, H, J) are analyzed on chase days 2 (A, B),4 (C, D), 7 (E, F), 10 (G, H) and 70 (I, J) for H2B-GFP epifluorescence (green) and DAPI (blue).
(TIF)

Figure S2 **Analysis of crypt-villus organoids derived from sorted LRCs.** Organoids encompass all differentiated intestinal lineages together with Lgr5+ CBCs. Serial sections from the same organoid were stained for HE (A), villin (B; brown; enterocytes), PAS and Alcian Blue (C: red, D; blue, both Goblet cells), lysozyme (E; brown; Paneth cells), and synaptophysin (F; brown; enteroendocrine cells). G. Lgr5 RT-PCR analysis. The upper and lower gel panels depict the products of Lgr5 and Actb RT-PCR analyses. Lanes 1 and 3: RNA from LRCs-derived organoids; lane 2: GFP–derived organoids;lane 4: organoids from whole crypts; lane 5: positive control; lane 6 negative control (no RNA input).
(TIF)

Figure S3 **Organoid-formation assay in Lgr5-EGFP mice.** (A) Organoid-forming capacity of sorted populations from Lgr5-EGFP mice. Displayed are average percentages of organoids formed (±s.e.m.) from 5000 FACS-sorted and plated events in 5 independent experiments as scored 14 days after plating. (B) To assess the presence of doublets among the sorted cells, the different subpopulations were examined by confocal microscopy. 500 cells were sorted in each well of a 6-well plate, imaged by confocal microscopy and counted. Representative IF images showing the composition of all sorted cell populations (left, - 20 µm) and their quantification by morphology for single cells and aggregates of differently sized cells (right), displayed as percentages.
(TIF)

Figure S4 **12Gy whole-body irradiation does not induce expression of the H2B-GFP transgene.** (A) Two sham pulse-chased (top) and two doxycycline pulse-chased (bottom) compound heterozygous villin-rtTA/TRE-H2B-GFP mice were subjected to 12 Gy whole-body irradiation and five injections of BrdU every four hours, following a twelve-hour recovery time after radiation. Animals were FACS-analyzed at 32 hours after radiation. (B) Results of the FACS analysis of live epithelial cells prepared from the intestines of these animals for GFP expression. Displayed are the average GFP-levels measured by FACS (± s.d.) 32 hours after radiation. While sham-pulsed control animals show only very low levels of GFP, doxycycline pulsed and chased animals display the clear presence of a GFP high and a GFP medium population. Doxycycline pulsed and chased animals have significantly more GFP medium cells than sham-pulsed mice (** p<0.001).
(TIF)

Figure S5 **Analysis of H2B-GFP retention following radiation treatment.** (A) The number of H2B-GFP nuclei per crypt was quantified for control mice, and on days 1, 2, 3, and 5 following radiation treatment. Graph shows increased numbers of LRC at days 3 and 5 (Day 3/5 v Day 0,** p value  = >0.001. Mann Whitney at least 75 crypts scored per day). (B) Quantification of the mean distance between the centers of H2B-GFP nuclei and the local center of the DICT layer. Graph shows that following radiation treatment H2B-GFP nuclei are located progressively more closely to the crypt base (Day 5 v Day 0, ** p value  = >0.001. Mann Whitney: at least 75 crypts scored per day). (C) The intensity of H2B-GFP signal is diluted on day 2 following radiation treatment. There is an 8 fold difference in the average intensity of nuclei A and B (within the same crypt). There is a 17 fold difference in the average intensity of nuclei B and C (within different crypts). Note that in order to visualize nucleus B, image contrast has been set so that both nuclei A and C appear saturated. Image field is 140×140 µm.
(TIF)

Figure S6 **Regeneration of the small intestine following tissue injury.** Compound heterozygous animals were subjected to tissue injury by 12 Gy of whole body ionizing irradiation at 14 days of chase. Two experimental animals were sacrificed at each 36 hours, 3 days, 4 days and 5 days following tissue injury. The morphology of the small intestine was analyzed by HE and immunohistochemistry for the intestinal differentiation markers PAS (goblet cells), synaptophysin (enteroendocrine cells) and lysozyme (Paneth cells). Complete regeneration following a radiation dosage of 12Gy is realized within the first four days. Cells of all the intestinal differentiation types are formed as shown by staining for PAS, synaptophysin and lysozyme at day 4 and 5. Crypt overgrowth recognizable by the appearance of elongated crypt structures can be observed at day 4 and 5 after irradiation.
(TIF)

Figure S7 **Methods. Description of the gates applied for the definition of singlets, doublets, higher level aggregates.** A preliminary gate (not shown) was applied on FSC-A vs SSC-A to discriminate cells from debris and big aggregates, followed by a gate to discriminate live and dead cells with hoechst 33258 (not shown). (A) A gate in FSC-H vs FSC-W was applied to divide singlets and doublets from higher level aggregates, and was refined by a similar gate in SSC-H vs. SSC-W (not shown). (B–D) The population of singlets + doublets was further analyzed for the pattern of expression of GFP (H2B-GFP LRCs and Lgr5-GFP) and CD24. (E–F) The selected populations were divided in singlets and doublets with a further gate on FSC-A vs SSC-A. The following populations were obtained: (G) CD24^h^iSSC^hi^ LRCs and GFP^-^CD24^h^iSSC^hi^ single cells, (I) Lgr5-GFP^hi^ singlets, (J) doublets of Lgr5-GFP^hi^ with CD24^hi^SSC^hi^ Paneth cells (GFP^+^CD24^hi^SSC^hi^). This strategy, based on the fine-tuning of the single cell gates on the specific populations of interest, allowed to obtain a higher percentage of single cells, together with a good recovery during sorting, if compared with a general initial stringent gate on FSC-H vs FSC-W (indicated by the dashed line in plot A).
(TIF)

Movie S1 **3D Multiphoton Microscopy of intestinal crypts from compound villin-rtTA/TRE-H2B-GFP animals after pulse (Movie S1, field size 300×300 µm).** After pulse, all nuclei within the crypt show H2B-GFP labeling (Movie S1).
(MOV)

Movie S2 **3D Multiphoton Microscopy of intestinal crypts from compound villin-rtTA/TRE-H2B-GFP animals following 36 days of chase (Movie S2, field size 250×250 µm).** Following 36 days of chase, LRCs (Movie S2, green nuclei) can be identified and localized within the entire volume of the lower third of the crypt. The collagen in the dense irregular layer of connective tissue (DICT) generates a strong signal when examined using Second Harmonic Generation (red) and thus acts as a robust standard to measure the distance of individual LRCs from the base of the crypt.
(MOV)

Table S1 **List of genes upregulated in EGFP^+^ cells (LRCs) when compared with EGFP^-^ cells.**
(XLSX)

Table S2 **Paneth cell-specific genes in LRCs collected after 46–47 days of chase.**
(XLSX)

Table S3 **Comparative analysis of genes differentially expressed in LRCs, (EGFP^+^/EGFP^-^) and in CD24^hi^SSC^hi^/Lgr5^+^.**
(XLSX)
